# Tryptophan synthase ß subunit 1 affects stomatal phenotypes in *Arabidopsis thaliana*

**DOI:** 10.3389/fpls.2022.1011360

**Published:** 2022-11-28

**Authors:** Midori N. Soda, Yuki Hayashi, Koji Takahashi, Toshinori Kinoshita

**Affiliations:** ^1^ Division of Biological Science, Graduate School of Science, Nagoya University, Chikusa, Nagoya, Japan; ^2^ Institute of Transformative Bio-Molecules (WPI-ITbM), Nagoya University, Chikusa, Nagoya, Japan

**Keywords:** stomata, guard cell, water loss, tryptophan biosynthetic pathway, tryptophan synthase ß subunit 1, PM H^+^-ATPase

## Abstract

Stomata open in response to several environmental stimuli, such as light and low CO_2_. Plasma membrane (PM) H^+^-ATPase in guard cells plays a pivotal role for light-induced stomatal opening. In contrast, stomata close in response to the dark or plant hormone abscisic acid (ABA). However, molecular mechanisms of stomatal movements remain unclear. To elucidate the molecular mechanism of stomatal movements, we performed a genetic screen based on stomatal aperture-dependent weight decrease of detached leaves from EMS-treated *Arabidopsis thaliana* and isolated a *rapid transpiration in detached leaves 2* (*rtl2*). The *rtl2* mutant showed constitutive open-stomata phenotype with lower leaf temperature. ABA had no effect on stomatal aperture in *rtl2*. The *rtl2* mutant also showed increased stomatal density, severe dwarf phenotype with pale green leaves and dark veins. Map-based analysis of the *RTL2* locus revealed that the *rtl2* mutant possesses a single nucleotide substitution, which induces amino acid substitution Gly162 to Glu in the tryptophan synthase ß subunit 1 (TSB1). The *TSB1* encodes an enzyme in tryptophan (Trp) biosynthetic pathway. Amount of TSB1 protein was drastically reduced in *rtl2* mutant. A different allele of *tsb1* mutant (*tsb1-1*) also showed constitutive open-stomata phenotype with reduced TSB1 protein as in *rtl2*. Analyses of test-crossed plants of *rtl2* and *tsb1-1* showed open-stomata and dwarf phenotypes. These results indicate that a responsible gene for *rtl2* is *TSB1*. We further investigated stomatal phenotype in mutants from Trp biosynthetic pathway, such as *wei2-1 wei7-1*, *trp3-1*, and *tsb2-1*. The *trp3-1* mutant showed significant wider stomatal aperture as well as *tsb1-1*. Trp biosynthetic pathway closely relates to auxin biosynthesis. Then, we investigated auxin responsible genes and found that an expression of *AUR3* was up in *rtl2*. In contrast, auxin had no effect on stomatal aperture in Arabidopsis and the phosphorylation status of PM H^+^-ATPase in guard cell protoplasts from *Vicia faba*. In addition, auxin antagonist had no effect on stomatal aperture. Interestingly, *tsb1-1* grown under hydroponic culture system showed normal stomatal aperture by exogenously application of Trp. These results suggest that open stomata phenotype in *tsb1-1* is due to Trp deficiency but not auxin.

## Introduction

Stomata in the plant epidermis, surrounded by a pair of guard cells, control gas exchange between the plants and the atmosphere. Opening of the stomata induces both transpiration and CO_2_ uptake for photosynthesis. Under drought condition, stomata close in response to the plant hormone abscisic acid (ABA) to prevent water loss (Schroeder et al., 2001; Shimazaki et al., 2007). Diverse external and internal stimuli, such as blue light (BL), red light (RL), the phytotoxin fusicoccin (FC), CO_2_, ABA, and microbial elicitors, regulate stomatal aperture (Munemasa et al., 2015; Inoue and Kinoshita, 2017; Ye et al., 2020). It has been demonstrated that stomatal opening contributes photosynthesis, plant growth, and yield (Wang et al., 2014; Toh et al., 2021; Zhang et al., 2021). In the BL-induced stomatal opening, BL photoreceptor phototropins activate plasma membrane (PM) H^+^-ATPase through the phosphorylation of C-terminal penultimate residue, threonine (Thr) (Kinoshita and Shimazaki, 1999; Kinoshita et al., 2001). BL-activated PM H^+^-ATPase creates an electrochemical gradient of H^+^ across the PM and an inside-negative electrical potential of PM that activate inward-rectifying voltage-gated K^+^ channels for K^+^ influx into guard cells (Schroeder et al., 1987). The K^+^ accumulation induces elevation of turgor pressure and opening of stomata (Schroeder et al., 2001; Shimazaki et al., 2007). Several signalling components, a protein kinase BLUE LIGHT SIGNALING1 (BLUS1) (Takemiya et al., 2013), a Raf-like protein kinase BLUE LIGHT-DEPENDENT H^+^-ATPASE PHOSPHORYLATION (BHP) (Hayashi et al., 2017), and a Type 1 protein phosphatase (PP1) (Takemiya et al., 2006), mediate the BL signalling pathway in guard cells. RL also induces stomatal opening through the phosphorylation of PM H^+^-ATPase penultimate residue, Thr, and activation of PM H^+^-ATPase in intact leaves (Ando and Kinoshita, 2018). However, the molecular mechanism of signalling pathway for light-induced stomatal opening remains incompletely understood (Inoue and Kinoshita, 2017).

Tryptophan (Trp) is an essential amino acid for all living organisms, but Trp is not synthesized in animals and some eubacteria. All catalytic enzymes and their encoding genes for Trp biosynthetic pathway have been identified in plants (Radwanski and Last, 1995; Tzin and Galili, 2010). The Trp biosynthesis includes six reaction steps from chorismate to Trp. In the first step, anthranilate synthase (AS) (CE 4.1.3.27) catalyzes a transfer of an amino group of glutamine to chorismate to generate anthranilate and pyruvate. In the second step, anthranilate phosphoribosylanthranilate transferase (PAT) (CE 2.4.2.1) catalyzes conversion of anthranilate and phosphoribosylpyrophosphate into phosphoribosylanthranilate and pyrophosphate. In the third step, phosphoribosylanthranilate isomerase (PAI) (CE 5.3.1.24) catalyzes conversion of phosphoribosylanthranilate into l-(O-carboxyphenylamino)-l-deoxyribulose-5-phosphate (CDRP). In the fourth step, indole-3-glycerol phosphate synthase (IGPS) (EC 4.1.1.48) catalyzes conversion of 1-(O-carboxyphenylamino)-1-deoxyribulose-5-phosphate to indole-3-glycerol phosphate. Trp synthase (TS) (CE 4.2.1.20), α (TSA) and ß (TSB) subunits, is involved in the last two steps. TSA cleaves indole-3-glycerol phosphate to indole and glyceraldehyde-3-phosphate. Finally, TSB catalyzes condensation of indole and serine to produce Trp. Trp biosynthetic pathway closely relates to auxin biosynthesis (Ouyang et al., 2000; Stepanova et al., 2008; Tao et al., 2008; Mashiguchi et al., 2011; Zhao, 2012; Tivendale et al., 2014).

Among them, Trp synthase ß subunit 1 (TSB1) is essential for Trp synthesis in plants; thus, it affects several developmental and physiological responses in plants (Last et al., 1991). Jing et al. (2009) reported that *smo1/trp2-301*, a mutant of *TSB1*, exhibits a reduction of the size of its aerial organs because of the retardation of growth by cell expansion, and that these phenotypes are rescued by addition of Trp. The *trp2* mutants also showed higher endogenous IAA content and increased expression of auxin responsive genes, such as *IAA1*, *IAA5* and *IAA6*. Ursache et al. (2014) reported that *trp2-12* and *trp2-13*, mutants of *TSB1*, showed incomplete vascular tissue development, and found that the expression of most of the HD-ZIPIII genes, which play a crucial role in xylem specification, is down regulated in the *trp2-1*. Wang et al. (2015) showed that *trp2-1*, a mutant of *TSB1*, has higher level of IAA than the wild type probably through the Trp-independent auxin biosynthetic pathway. Liu et al. (2022) reported that TSB1 interacts with ABA metabolism enzyme, ß-glucosidase 1 and mediates regulation of plant growth and abiotic stress responses.

In the previous study, we performed a screen focused on stomatal aperture-dependent of weight decrease of the detached leaves from ethyl methanesulfonate (EMS)-treated *Arabidopsis thaliana* and identifed a *rapid transpiration in detached leaves 1* (*rtl1*) mutant. The *rtl1* mutant showed ABA-insensitive stomatal phenotype and possesses a novel missense mutantion in the Mg-chelatase H subunit (CHLH). It is worthy of note that CHLH affects ABA-induced stomatal closure and inhibition of stomatal opening, but does not act as an ABA receptor (Tsuzuki et al., 2011). In this study, we isolated *rtl2* mutant, which shows open-stomata phenotype. Map-based analysis and genetic analysis strongly indicated that a responsible gene for *rtl2* is *TSB1* involved in Trp biosynthetic pathway. Based on phenotypic and genetic analyzes, we propose that the Trp synthesis pathway has a significant effect on stomatal phenotypes, control of stomatal aperture and density.

## Materials and methods

### Plant materials and growth conditions

For plant growth on soil, plants were grown under 16-h fluorescent light (50 µmol m^-2^ s^-1^)/8-h dark cycle at 24°C in 55-70% humidity in a growth room. For plant growth on plate followed by on soil, seeds were surface-sterilized and sown on Murashige and Skoog-agar plate supplied with 1% (w/v) sucrose under 16-h fluorescent light (50 µmol m^-2^ s^-1^)/8-h dark cycle. Four-week-old plants were transferred to soil and grown under 16-h fluorescent light (50 µmol m^-2^ s^-1^)/8-h dark cycle at 24°C in 55-70% humidity in a growth room. *gl1* [Columbia (Col-0), carrying *gl1* mutation] is the background ecotype of an *rtl2* mutant and used as the wild type (WT). We backcrossed *rtl2* with *gl1* three times. The mutants used in this study, *tsb1-1* (SAIL_886_A01), *tsb2-1* (SAIL_598_H05), *trp3-1* (CS8331; Radwanski et al., 1996), *wei2-1 wei7-1* (CS16399; Stepanova et al., 2005) were obtained from the Arabidopsis Biological Resource Center (ABRC) (Ohio State University, Columbus, OH, USA). Col-0 is the background ecotype of these mutants. For plant growth in hydroponic culture, seeds were surface-sterilized and sown on Murashige and Skoog-agar plate as described above. Ten-day-old seedlings were transferred to hydroponic culture system with a nutrient solution (Gibeaut et al., 1997). The solution was constantly replaced every 1 week.

### Isolation of the *rtl2* mutant and identification of the *RTL2* locus

Mutant screening based on stomatal aperture-dependent water loss was performed as previously described (Tsuzuki et al., 2011). Briefly, ethyl methanesulfonate (EMS)-treated *gl1* M_2_ seeds were germinated and grown on soil. We measured the fresh weight of a detached rosette leaf at 0 and 90 min from each 4-week-old M_2_ plant and isolated some *rapid transpiration in detached leaves* (*rtl*) mutants (Tsuzuki et al., 2011). In this study, we investigated an *rtl2* mutant, which shows rapid weight change compared to WT plants. Mapping populations were generated by crossing the *rtl2* mutant with the Landsberg *erecta* (L*er*) accession of *Arabidopsis thaliana*. *RTL2* locus was identified by mapping using simple-sequence length polymorphism (SSLP) and cleaved amplified polymorphism (CAPS) markers from 910 F_2_ plants showing dwarf phenotype with pale green leaves and dark veins.

### Measurement of stomatal aperture

Stomatal apertures in the isolated epidermis were measured according to the previous method (Inoue et al., 2008) with modifications. The epidermal fragments isolated from overnight dark-adapted 4- to 6-week-old plants or 4- to 6-week-old plants from illuminated growth condition at zeitgeber time (ZT) 4 to 7 were incubated in basal buffer (5 mM MES-BTP, pH 6.5, 50 mM KCl, and 0.1 mM CaCl_2_). For investigations of light-induced stomatal opening and effect of ABA or auxinole, the epidermal fragments were incubated under light [blue light (Stick-B-32; EYELA, Tokyo, Japan) at 10 µmol m^-2^ s^-1^ superimposed on background red light (LED-R; EYELA, Tokyo, Japan) at 50 µmol m^-2^ s^-1^] at 24°C in the presence or absence of 20 µM ABA or 10 µM auxinole for 2.5 hr or kept in the dark at 24°C for 2.5 hr. For investigation of the effect of auxin, the epidermal tissues were incubated in the basal buffer for 1.5 hr in darkness to deplete endogenous auxin. Then, the pre-incubated epidermal tissues were treated with 10 µM IAA in the dark for 3 hr. Stomatal apertures were measured microscopically by focusing on the inner lips of stomata in the abaxial epidermis.

For determination of stomatal aperture from test-crossed plants under illuminated growth condition, we used intact leaves for measurements according to the previous method (Toh et al., 2018). Rosette leaves were detached from the plants at ZT 4 to 7. We cut out central areas of the leaves without the midrib were cut out and mounted leaf disc on microscope slides with the abaxial side attached to the cover glass. We obtained Images using an optical microscope (BX43; Olympus) with a charge-coupled device (CCD) camera (DP27; Olympus) with a x20 objective lens (UPlanFL N; Olympus). For getting extended focus imaging, we used cellSens standard software (Olympus) to maximize the number of analyzable focused stomata within each image. Stomatal apertures in the abaxial side were measured on the inner lips of stomata using ImageJ software (http://imagej.nih.gov/ij/).

### Determination of leaf temperature using the infrared thermography

Plants were grown on MS plate for 4 weeks and transferred to soil in each pot for 1 week. We measured leaf temperatures using a TVS-500EX infrared thermography instrument (NEC Avio Infrared Technologies Co., Ltd.) and analyzed images using the Avio Thermography Studio software.

### Determination of stomatal size and density

Stomatal size and density were determined according to a previous method (Wang et al., 2011).

### RT-PCR and quantitative RT-PCR

We purified total RNA from rosette leaves of 4- to 6-week-old plants using an RNeasy Plant Mini Kit (Qiagen). We synthesized 1st-strand cDNAs using a Takara PrimeScript II First Strand cDNA Synthesis Kit (Takara) with oligo(dT)_12–18_ primer. For RT-PCR, we amplified cDNA fragments by PCR using specific primers (**Supplementary Table S2**). Quantitative RT-PCR (qRT-PCR) was performed as previously described (Kinoshita et al., 2011) using specific primers (**Supplementary Table S2**). We used *TUB2* (AT5G62690) as an internal standard for PCRs.

### Anti-TSB1 antibody

Anti-TSB1 antibody was raised against the recombinant TSB1ΔN, TSB1 protein without chloroplast targeting signal peptide in its N-terminus, as an antigen in a rabbit (Medical & Biological Laboratories). We amplified the *TSB1* DNA fragment from 1st-strand Arabidopsis cDNA by PCR using the specific primers 5′-CGGGATCCGACCCGGCCCTGTGGCAAC-3′ and 5′-CGGGATCCTCAAACATCAAGATATTTAGCCACTGTCTGAAC-3′. We cloned the *TSB1* CDS of 202–1413 bp containing *Bam*HI site at both ends into the *Bam*HI site of the pGEX-2T vector (GE Healthcare) to express as a fusion protein with glutathione S-transferase (GST). The pGEX-2T-TSB1 was transformed into the *E. coli* BL21 strain. The fusion protein (GST-TSB1ΔN) was purified using the glutathione-Sepharose 4B (GE Healthcare). The TSB1ΔN protein was obtained by digestion with thrombin to cut off the GST and used for the immunization as antigen.

### Immunoblots

Immunoblot analysis was performed according to the methods described in Hayashi et al. (2010) with modifications. We grinded leaves from 5- to 6-week-old plants using a mortar and pestle in extraction buffer (50 mM MOPS–KOH, pH 7.5, 2.5 mM EDTA, 100 mM NaCl, 1 mM phenylmethylsulfonyl fluoride, 20 µM leupeptin, and 2 mM DTT). Proteins (20 µg) were loaded and separated by SDS-PAGE. We detected TSB1 protein using the polyclonal antibody raised against recombinant TSB1ΔN protein (Anti-TSB1 antibody) in rabbits. We detected the 14-3-3 proteins with the anti-14-3-3 protein (GF14 ø) antibody (Kinoshita and Shimazaki, 1999) as a control. We used the antibodies at a 3,000-fold dilution.

Detections of PM H^+^-ATPase protein and phosphorylation status of the penultimate residue, Thr, of PM H^+^-ATPase in guard cell protoplasts (GCPs) from *Vicia faba* were performed using anti-H^+^-ATPase antibody and anti-pThr antibody, respectively (Hayashi et al., 2010).

### Measurement of Tryptophan synthase ß subunit activity

TSB activity was determined according to Greenberg and Galston (1959) with modifications. For measurement in leaf extracts, leaves from 5- to 6-week-old plants were ground in phosphate buffer (0.1 M Potassium phosphate-KOH, pH 8.2, 30% [w/v] Insoluble-Polyvinylpolypyrrolidone [Sigma-Aldrich]) with 212-300 µm glass beads (Sigma-Aldrich) using a mortar and pestle. Then the extract was sonicated and centrifuged at 4 °C (12,000 *g* for 15 min). The supernatant was used as the enzyme source. The supernatant (150–250 µg of protein) was suspended in reaction buffer (50 µl; 80 mM Potassium phosphate-KOH, pH 8.2, 60 mM L-Serine, 200 µM indole, 10 µg/ml pyridoxal phosphate) and reacted at 30 °C for 90 min. The reaction was stopped by the addition of 5 µl of 0.2 M NaOH. To assay for residual unreacted indole, 200 µl of toluene was added to the reaction mixture and then shaken and centrifuged at 1,500 *g* for 15 min. The resulting toluene layer was transferred and mixed with 4 times the volume of Ethanol and twice the volume of Ehrlich’s reagent (36 mg/ml *p*-dimethylaminobenzaldehyde, 2.13 M HCl dissolved in Ethanol). After incubation at 25 °C for 30 min, the absorbance at 540 nm was measured and differences of absorbance between before and after the reaction was converted to the disappearance of indole using the standard curve generated with dilution series of indole. For expression of recombinant TSB1ΔN protein, the *TSB1* DNA fragments derived from WT and *rtl2* were cloned into the vector as described above. The recombinant TSB1ΔN proteins were purified using the glutathione-Sepharose 4B (GE Healthcare) and used for measurement of TSB activity.

### Isolation of GCPs from *Vicia faba* and auxin treatments

We isolated guard cell protoplasts (GCPs) enzymatically from lower epidermis of leaves from 4- to 6-week-old *Vicia faba* according to a previous method (Hayashi et al., 2021). GCPs in suspension buffer (5mM MES-NaOH [pH 6.0], 10mM KCl, 0.4 M mannitol and 1mM CaCl_2_) were treated with auxins (IAA, 1-NAA, 2,4-D) and fusicoccin (FC) at 10 µM in the dark for 30 min. Proteins (20 µg) were loaded and separated by SDS-PAGE.

### Accession number

ASA1; AT5G05730, ASB1; AT1G25220, AUR3; AT4G37390, IAA1; AT4G14560, IAA24; AT4G14560, SAUR9; AT4G36110, TSA1; AT3G54640, TSB1; AT5G54810, TSB2; AT4G27070, TUB2; AT5G62690.

## Results

### An *rtl2* mutant exhibited widely opened stomatal phenotype

**Figure 1:**
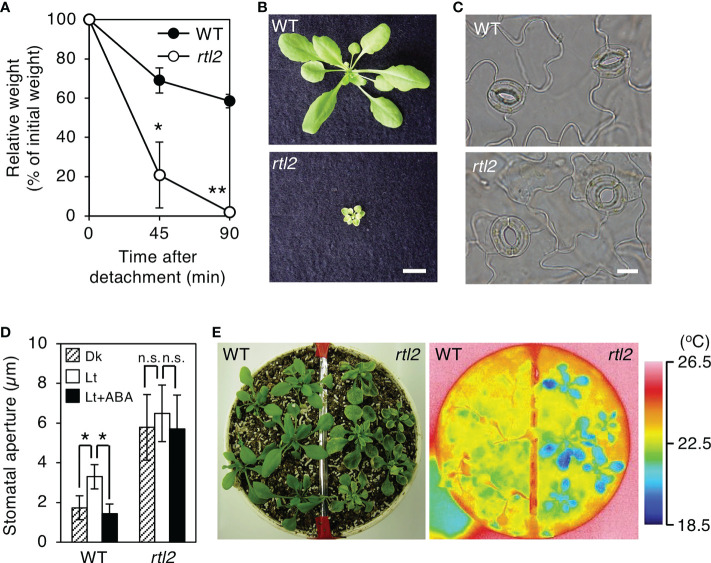
Characterization of the *rtl2* mutant. **(A)** Kinetics of the fresh weight change in the detached rosette leaves from 4-week-old WT (closed circles) and *rtl2* (open circles) plants. The relative weights of leaves are presented as a percentage of the initial weight, which was the weight of each rosette leaf immediately after detachment from the plants. Values are means of three independent experiments with SDs. Asterisks indicate a significant difference in leaf weight change relative to WT by Student *t* test; *P < 0.05, **P < 0.01. **(B)** Four-week-old WT and *rtl2* plants grown on soil. Scale bar = 1 cm. **(C)** Typical stomata in the epidermis of WT and *rtl2* plants under illuminated growth conditions. Scale bar = 10 µm. **(D)** Stomatal aperture of 5-week-old WT and *rtl2* plants. Plants were grown on MS plate for 4 weeks and transferred to soil for 1 week. Epidermal tissues from dark-adapted plants were incubated under light (blue light at 10 µmol m^-2^ s^-1^ superimposed on red light at 50 µmol m^-2^ s^-1^) with (black; Lt+ABA) or without (white; Lt) 20 µM ABA or kept in the dark (hatched; Dk) for 2.5 hr. Values represent mean ± SD (n = 3); measurement of > 25 stomata in each experiment. Asterisks indicate a significant difference by Student *t* test; *P < 0.05. n.s., not significant. **(E)** Thermal image (right) and the corresponding bright-field image (left) of WT and *rtl2* plants. The images of the 5-week-old plants were grown on MS plate for 4 weeks and transferred to soil for 1 week.

To elucidate the mechanism of stomatal movement, we have performed a mutant screening based on stomatal aperture-dependent weight loss of detached leaves *via* transpiration using a microbalance (Tsuzuki et al., 2011). In addition to a *rapid transpiration in detached leaves 1* (*rtl1*) mutant (Tsuzuki et al., 2011), we isolated an *rtl2* mutant. The *rtl2* showed rapid weight loss of detached leaves under growth condition. The WT (Col-*gl1*; background plant of the screening) leaf weight decreased to 59% of initial weight for 90 min, whereas the *rtl2* leaf weight decreased to 2% (**Figure 1A**). In addition, *rtl2* mutant showed severe dwarf phenotype with pale green leaves and dark veins under soil grown condition (**Figure 1B**). It is worthy of note that stomatal size and index in *rtl2* and *tsb1-1* were almost comparable to those in background strains. In contrast, stomatal density in *rtl2* and *tsb1-1* were ~30-46% increase compared to that in background strains (**Table 1**). Furthermore, color of dry seeds from *rtl2* and *tsb1-1* showed a lighter color than background strain seeds (**Supplementary Figure 1**) (Last et al., 1991).

**Table 1:** Stomatal size, index and density in plants.

	Stomatal size (µm)	Stomatal index	Stomatal density (mm^-2^)
WT	22.7 ± 0.81	0.267 ± 0.036	94.8 ± 24.2
*rtl2*	21.9 ± 1.38*	0.254 + 0.057	123 ± 24.8**
Col-0	22.5 ± 1.09	0.243 + 0.033	107 ± 24.3
*tsb1-1*	22.2 ± 1.13	0.210 + 0.033*	156 ± 54.4**

Stomatal size, index, and density were calculated according to a previous method (Wang et al., 2011). Asterisks indicate a significant statistical difference relative to background plants by Student t test (*P < 0.05, **P < 0.01).

As shown in **Figure 1C**, stomata of *rtl2* opened widely compared to WT under illuminated growth condition. We further examined stomatal responses of *rtl2* in detail. The stomata in WT closed in the dark and opened in response to light, and ABA (20 µM) suppressed light-induced stomatal opening (**Figure 1D**). By contrast, the stomata in *rtl2* opened widely even in the dark and 20 µM ABA had no effect on stomatal aperture. In accord with open-stomata phenotype in *rlt2* mutant, *rtl2* mutant exhibited clear low leaf temperature phenotype under illuminated growth condition (**Figure 1E**). Average leaf temperature of the *rtl2* mutant was reduced over 3.0°C relative to WT.

### Open stomata phenotype is caused by a missense mutation in *TSB1*

**Figure 2:**
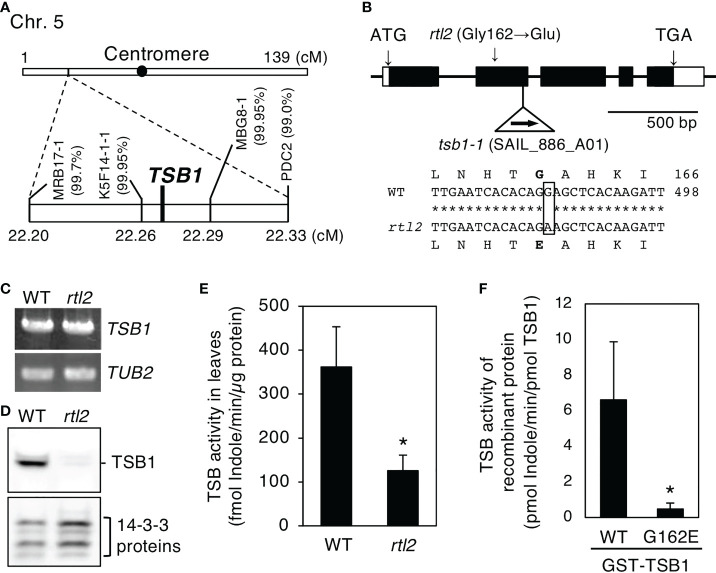
Determination of the mutation in the *rtl2* mutant. **(A)** Mapping analysis of the *RTL2* locus. Numbers in parentheses indicate percentages of no recombination in 1820 chromosomes. The *RTL2* locus was close to CAPS marker K5F14-1-1, MBG8-1 and *Tryptophan synthase ß subunit 1* (*TSB1*). **(B)** Schematic representation of the structure of the *TSB1* gene and the position of T-DNA insertion in the *tsb1-1* mutant (upper). Boxes and lines represent exons and introns respectively. The T-DNA insertion was located in 2nd exon of the *TSB1* gene. The position of the amino acid substitution (Gly162 to Glu) in *rtl2* is indicated. The partial sequences of *TSB1* cDNA and the deduced amino acid in WT and *rtl2* are shown (lower). A single nucleotide substitution (G485 to A) is shown by a box. Nucleotide and amino acid numbers are indicated in the right. Asterisks indicate the same nucleotide of the *TSB1* gene in WT and *rtl2*. **(C)** *TSB1* expression analyzed by RT-PCR in WT and *rtl2*. Total RNA was extracted from rosette leaves of 5-week-old plants grown on MS plate for 4 weeks and transferred to soil for 1 week. *TUB2* was amplified as a control. PCRs were performed with 30 cycles for *TSB1* and with 25 cycles for *TUB2*, respectively. **(D)** Immunoblot analysis of TSB1 protein in WT and *rtl2*. Twenty micrograms of protein extracted from rosette leaves of 5-week-old plants was loaded on each lane. The 14-3-3 proteins were detected using anti-14-3-3 antibody as a control. **(E)** TSB activity in rosette leaves of 5-week-old WT and *rtl2*. Values are means of three independent experiments with SDs. Asterisk indicates a significant difference in TSB activity relative to WT by Student *t* test (*P < 0.05). **(F)** TSB activity of recombinant TSB1 (WT) and TSB1-G162E. Values are means of three independent experiments with SDs. Asterisk indicates a significant difference in TSB activity relative to WT by Student *t* test (*P < 0.05).

To identify the *RTL2* locus, we performed a map-based analysis and found strong linkage the CAPS marker K5F14-1-1 and MBG8-1 in *rtl2* (**Figure 2A**). According to the Arabidopsis Information Resource (TAIR) database, *Tryptophan synthase beta-subunit 1* (*TSB1*; AT5G54810) is a candidate gene. Because there is a *TSB1* locus between K5F14-1-1 and MBG8-1 and known *TSB1* mutants showed dwarf phenotype with pale green leaves and dark veins same as in *rtl2* (**Figures 1B**, **2B**) (Jing et al., 2009). Sequence analysis of *TSB1* cDNA from *rtl2* revealed a single nucleotide substitution from G485 to A, which induces a novel missense mutation from Gly162 to Glu (**Figure 2B**). Next, we investigated the*TSB1* transcript level and protein amount of TSB1 in rosette leaves. As shown in **Figure 2C, 2D**, the level of *TSB1* transcript was similar to that of the WT, whereas the amount of TSB1 protein was significantly decreased in *rtl2*. We further determined the TSB activity in leaf extracts by measuring disappearance of indole. The results showed that *rtl2* has ~35% TSB catalytic activity compared to that of WT (**Figure 2E**). Reduction of TSB activity in *rtl2* may be caused by not only lower amount of TSB1 protein but also less activity of *rtl2* mutation (Gly162 to Glu in TSB1), because the recombinant TSB1-G162E has only ~7.2% TSB activity compared to that of WT-TSB1 (**Figure 2F**).

**Figure 3:**
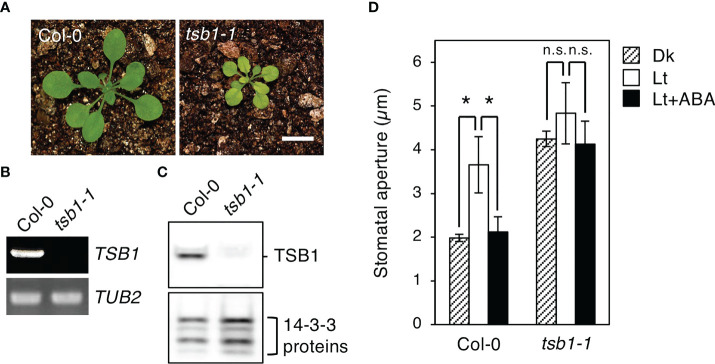
Phenotypic analysis of a *TSB1* knockout mutant, *tsb1-1*. **(A)** Five-week-old Col-0 and *tsb1-1* plants. Plants were grown on MS plate for 4 weeks and transferred to soil for 1 week. Scale bar = 1 cm. **(B)** *TSB1* expression analyzed by RT-PCR in Col-0 and *tsb1-1*. Other details are the same as in Figure 2C. **(C)** Immunoblot analysis of TSB1 protein in Col-0 and *tsb1-1*. Other details are the same as in Figure 2D. **(D)** Stomatal aperture of Col-0 and *tsb1-1* plants. Other details are the same as in Figure 1D. Values represent mean ± SD (n = 3); measurement of 35 stomata in each experiment. Asterisks indicate a significant difference by Student *t* test; *P < 0.05. n.s., not significant.

**Figure 4:**
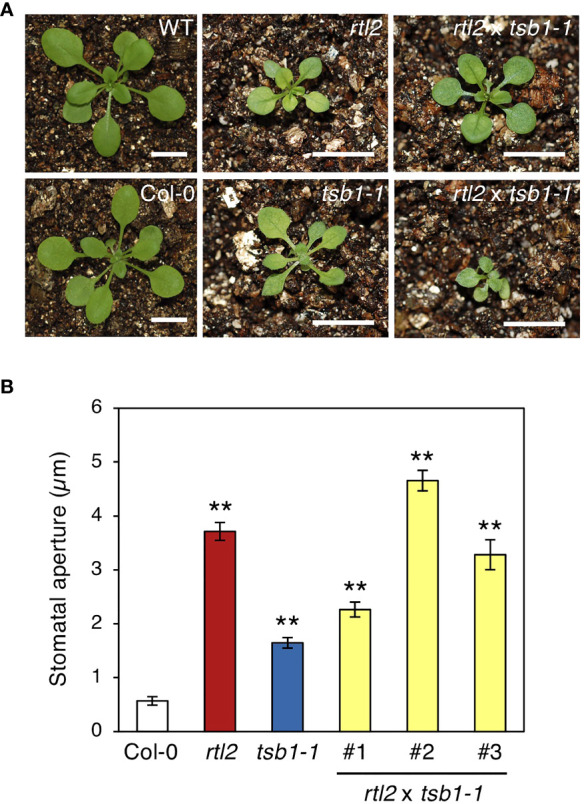
Phenotypes of test-crossed plants. **(A)** Five-week-old WT, Col-0, *rtl2*, *tsb1-1* and test-crossed F_1_ plants (*rtl2* x *tsb1-1*). Plants were grown on MS plate for 4 weeks and transferred to soil for 1 week. The scale bar represents 1 cm. **(B)** Stomatal aperture of 5-week-old Col-0, *rtl2*, *tsb1-1* and test-crossed F_1_ plants (*rtl2* x *tsb1-1*) under illuminated growth condition. Rosette leaves were detached from the plants at ZT4-7 and immediately subjected to measurement of stomatal aperture. Values are means of > 25 stomata with SDs. Asterisks indicate a significant statistical difference relative to Col-0 by Student *t* test (**P < 0.01).

To determine whether *rtl2* is an allele of *tsb1*, a T-DNA insertion mutant *tsb1-1* (SAIL_886_A01) was obtained from ABRC (**Figure 2B**). The *tsb1-1* showed *rtl2*-like dwarf phenotype with pale green leaves and dark veins (**Figure 3A**). RT-PCR analysis revealed that *tsb1-1* is a knockout mutant, which results in significant decrease of protein amount in rosette leaves of *tsb1-1* (**Figures 3B, C**). Stomata of *tsb1-1* also opened widely even in the dark and could not close in response to ABA, suggesting that *TSB1* is responsible for the *rtl2* phenotype (**Figure 3D**). To confirm this, we have tried to complement the WT *TSB1* genome to *rtl2*, however, we could not obtain the transgenic *rtl2* plants carrying the WT *TSB1* gene under its own promoter, probably due to growth defect of *rtl2* plants. Therefore, we performed test-cross of *rtl2* and *tsb1-1* to show that these are allelic gene. As shown in **Figure 4A**, F_1_-generation plants showed dwarf phenotype with pale green leaves and dark veins same as in *rtl2* and *tsb1-1*, indicating that these are allelic gene. In addition, stomata of F_1_-generation plants widely opened under illuminated growth condition (**Figure 4B**). From these results, we concluded that *TSB1* is responsible for the *rtl2* phenotype.

### Mutant of another tryptophan synthetic enzyme *TSA1* also had open stomata phenotype

**Figure 5:**
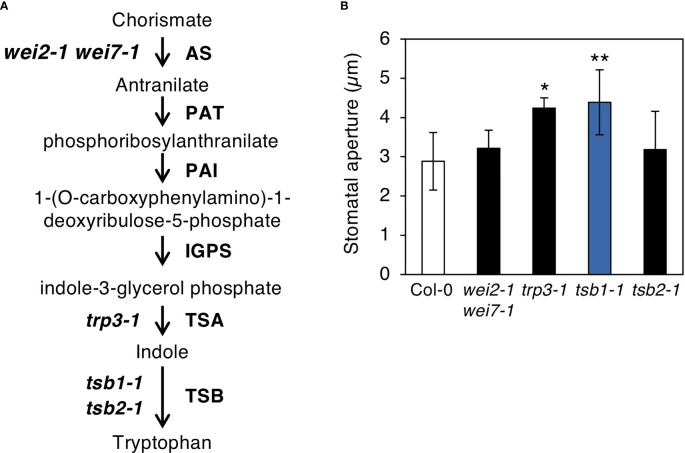
Characterization of the mutants related to Tryptophan biosynthetic pathway. **(A)** Tryptophan biosynthetic pathway in *Arabidopsis thaliana*. Tryptophan biosynthetic pathway starting from chorismate are shown with the enzymes of each step (right) and the mutants used in this study (left). AS, anthranilate synthase; PAT, anthranilate phosphoribosylanthranilate transferase; PAI, phosphorybosylanthranilate isomerase; IGPS, indole-3-glycerol phosphate synthase; TSA, tryptophan synthase α subunit; TSB, tryptophan synthase ß subunit. **(B)** Stomatal aperture of Tryptophan biosynthetic mutants under illuminated growth condition. Epidermal tissues were isolated from 5-week-old plants grown on MS plate for 4 weeks and transferred to soil for 1 week at ZT4-7 and immediately subjected to measurement of stomatal aperture. Values represent mean ± SD (n = 3); measurement of > 25 stomata in each experiment Asterisks indicate a significant statistical difference relative to Col-0 by Student *t* test (*P < 0.05, **P < 0.01).

TSB1 encodes a predominantly expressed Trp synthase ß subunit in Trp biosynthetic pathway, in which Trp is synthesized from chorismate *via* six reaction steps as shown in **Figure 5A** (Radwanski and Last, 1995; Tzin and Galili, 2010). To investigate whether other Trp biosynthesis enzymes affect stomatal opening, we measured the stomatal aperture of the double mutant of AS, *wei2-1 wei7-1*, single substitution mutant of TSA1, *trp3-1*, and a T-DNA insertion knockout mutant of TSB1 paralog, TSB2 (*tsb2-1*; **Supplementary Figure S2**). Among the mutants, *trp3-1* showed open stomata phenotype similar to *tsb1-1*, but not in *wei2-1 wei7-1* and *tsb2-1* (**Figure 5B**).

### Auxin treatment did not affect stomatal aperture

**Figure 6:**
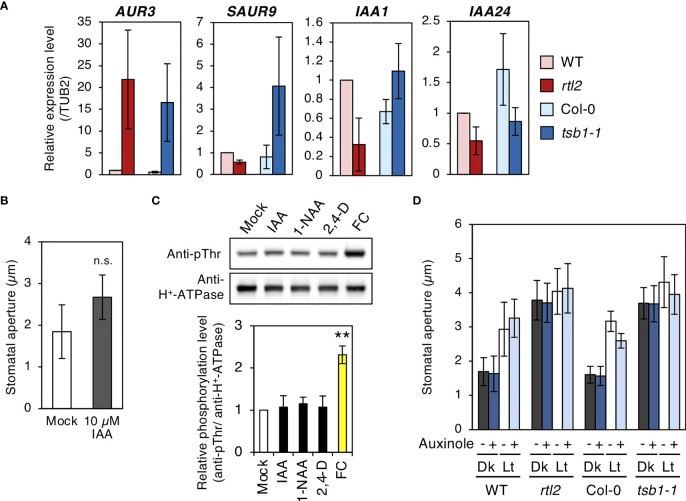
Characterizations of *rtl2* and *tsb1-1* mutants and auxin responses in stomata. **(A)** Expression of auxin responsive genes analyzed by qRT-PCR in WT, *rtl2*, Col-0 and *tsb1-1*. Total RNA was extracted from rosette leaves of 5-week-old plants grown on MS plate for 4 weeks and transferred to soil for 1 week. *TUB2* was amplified as a control. **(B)** Effect of exogenous auxin on stomatal aperture in Col-0. Epidermal tissues from dark-adapted 4-week-old plants grown on soil were incubated with 0.25% (v/v) DMSO (Mock) and10 µM IAA in the dark for 3 hr. Values represent mean ± SD (n = 4); measurement of 45 stomata in each experiment n.s. not significant. **(C)** Effect of auxins to the phosphorylation status of the penultimate residue, Thr, of PM H^+^-ATPase in guard cell protoplasts (GCPs) from *Vicia faba*. GCPs were incubated with IAA and synthetic auxins (1-NAA, 2,4-D) and fusicoccin (FC) at 10 µM in the dark for 30 min. DMSO was used as a solvent control (Mock). Immunoblots of the phosphorylated PM H^+^-ATPase and total PM H^+^-ATPase were performed using anti-pThr antibody and anti-H^+^-ATPase antibody, respectively. The graph below the blot shows the relative phosphorylation level of PM H^+^-ATPase, defined as the ratio of the phosphorylated PM H^+^-ATPase to the total quantity of the protein, set as 1 for Mock. The bars represent the means ± SD for three independent experiments. Asterisks indicate a significant statistical difference relative to Col-0 by Student *t* test (**P < 0.01). **(D)** Effect of auxin antagonist, auxinole, on light-induced stomatal opening in WT, *rtl2*, Col-0 and *tsb1-1*. Plants were grown on MS plate for 4 weeks and transferred to soil for 1 week. Epidermal tissues from dark-adapted plants were incubated under light (Lt) or kept in the dark (Dk) in the presence or absence of auxinole for 2.5 hr. Other details are the same as in **Figure 1D**.

Indole-2-glycerol phosphate and Indole are substrates of TSA and TSB respectively. These are thought to be converted to indole-3-acetic-acid (IAA) *via* Trp-independent pathway (Ouyang et al., 2000). Jing et al. (2009) reported that free IAA levels in two *tsb1* alleles, *trp2-1* and *trp2-8*, were elevated and auxin responsive genes were up-regulated in these mutants. To clarify whether IAA contents in *rtl2* is also elevated, we investigated the expression level of some auxin responsive genes, *AUXIN UP REGULATED 3* (*AUR3*), *SMALL AUXIN UPREGULATED RNA 9* (*SAUR9*), *INDOLE-3-ACETIC ACID INDUCIBLE 1* (*IAA1*) and *INDOLE-3-ACETIC ACID INDUCIBLE 24* (*IAA24*). As shown in **Figure 6A**, *AUR3* was significantly up-regulated in both *rtl2* and *tsb1-1*, though other auxin responsive genes were not. Given that up-regulation of *AUR3* expression in *tsb1* alleles, we speculated that open stomata phenotype in *rtl2* is caused by elevated levels of auxin. To address the possibility, we first investigated the effect of auxin on stomatal aperture. Incubation of the epidermal tissues with 10 µM IAA in the dark for 3 hr had no effect on stomatal aperture (**Figure 6B**). Next, we investigated effect of auxins, IAA and synthetic auxins, 1-naphthylacetic acid (1-NAA) and 2,4-dichlorophenoxyacetic acid (2.4-D), on phosphorylation status of the penultimate residue, Thr, of PM H^+^-ATPase in guard cell protoplasts (GCPs) from *Vicia faba*, because auxin activates PM H^+^-ATPase *via* phosphorylation during auxin-induced hypocotyl elongation (Takahashi et al., 2012; Uchida et al., 2018). However, consistent with a recent report that IAA did not induce phosphorylation of PM H^+^-ATPase in guard cells of *Arabidopsis thaliana* (Akiyama et al., 2022), all auxins had no effect on phosphorylation status of PM H^+^-ATPase for 30 min, although a fungal toxin fusicoccin (FC) induced phosphorylation of PM H^+^-ATPase (**Figure 6C**). These results suggest that short term treatment of auxin (within 3 hr) has no effect on both stomatal aperture and phosphorylation status of PM H^+^-ATPase in guard cells. Furthermore, we found that auxin antagonist, auxinole (Hayashi et al., 2012), did not change the stomatal aperture in *rtl2* and *tsb1-1* (**Figure 6D**).

### Exogenous Trp rescued the phenotypes of *tsb1-1*

**Figure 7:**
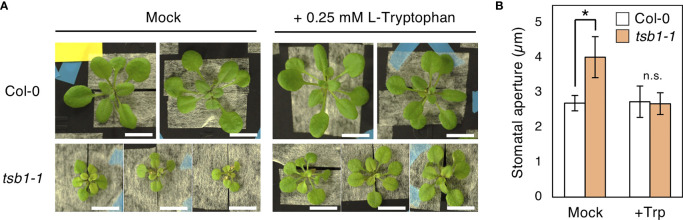
Effect of exogenous L-Trp on the phenotypes of *tsb1-1*. **(A)** Four-week-old Col-0 and *tsb1-1* plants grown with or without L-Trp. Plants were grown on MS plate for 10days and transferred to hydroponic system with or without 0.25 mM L-Trp for 2 weeks. Scale bar = 1 cm. **(B)** Stomatal aperture of Col-0 and *tsb1-1* plants grown with or without L-Trp under illuminated growth condition. 10-day-old seedlings germinated on MS plate were transferred to hydroponic system with or without 0.25 mM L-Trp, and were grown for a further 3 to 4 weeks. Epidermal tissues were isolated at ZT4-7 and immediately subjected to measurement of stomatal aperture. Values represent mean ± SD (n = 3); measurement of > 25 stomata in each experiment. Asterisk indicates a significant statistical difference relative to Col-0 by Student *t* test (*P < 0.05; n.s., not significant).

To verify whether the open stomata phenotype in *tsb1* mutants is caused by Trp deficiency, L-Tryptophan (L-Trp) was exogenously supplied to *tsb1-1*. We used only *tsb1-1* mutant in this experiment due to less growth of *rtl2* in hydroponic culture. Ten-day-old *tsb1-1* seedlings grown on MS plate were transferred to hydroponic system supplemented with/without 0.25 mM L-Trp for 2-4 weeks. Similar to the previous report (Jing et al., 2009), dwarf and pale green phenotype in *tsb1-1* were partially rescued by exogenous application of L-Trp (**Figure 7A**). Interestingly, *tsb1-1* showed normal stomatal aperture by application of L-Trp (**Figure 7B**), suggesting that open stomata phenotype in *tsb1-1* is due to Trp deficiency. On the other hand, under hydroponic culture condition, stomatal density in *tsb1-1* did not show significant differences compared to that in Col-0 and L-Trp application did not change the stomatal density in both Col-0 and *tsb1-1* (**Supplementary Table S1**). Therefore, Trp deficiency may have no effect on the stomatal density.

## Discussion

In this study, we isolated *rapid transpiration in detached leaves 2* (*rtl2*), that exhibited a higher rate of water loss *via* transpiration and clear low leaf temperature phenotype (**Figures 1A, E**). Detailed analysis of stomatal phenotypes of *rtl2* revealed that constitutive open-stomata phenotype and higher stomatal density contribute rapid transpiration in *rtl2* (**Figure 1D**, **Table 1**). Map-based analysis and test-cross with *tsb1-1* revealed that *Tryptophan synthase ß subunit 1* (*TSB1*) is responsible for *RTL2* locus (**Figures 2**, **3**, **4**). The missense mutation of TSB1 Gly162 to Glu found in *rtl2* resulted in significantly reduction of TSB activity (**Figure 2F**). Even though the transcript level of *TSB1* in *rtl2* were comparative to that in WT, amount of TSB1 protein in *rtl2* was significantly reduced (**Figures 2C, D**). This could be due to active degradation of mutated TSB1 in *rtl2*. The residual TSB activity in *rtl2*, ~35% of WT, may come from *TSB2*, a paralog of *TSB1* (**Figure 2E**).

Very recently, Liu et al. (2022) reported that *amiR*-TSB1 lines, which have reduced expression of *TSB1* to 64-73% of wild-type plants, exhibited lower water loss rate from detached leaves and higher survival rate under salt and drought stress compared with wild-type plants. They also found that TSB1 interacts with and inhibits *ß*-glucosidase (BG1), catalyzing the conversion of glucose-conjugated ABA into active ABA. In consistent with this, the ABA contents in *amiR*-TSB1 lines were significantly higher than those in the wild-type. They concluded that TSB1 regulates stress tolerance and the accumulation of ABA *via* repression of BG1 activity. In contrast, we found that stomata in two *tsb1* mutants, *rtl2* and *tsb1-1*, significantly opened even in the dark condition and in the presence of ABA (**Figures 1**, **3**). Thus, since the phenotype related to stomatal aperture is considered to be greatly different between *amiR*-TSB1 lines and *rtl2* and *tsb1-1*, it is possible that *amiR*-TSB1 lines induce salt and drought resistance regardless of stomatal phenotype. Therefore, *rtl2* and *tsb1-1* may exhibit such salt and drought tolerance, it is necessary to investigate the tolerance in *rtl2* and *tsb1-1*. As a point of concern, the amount of protein in *amiR*-TSB1 lines have decreased to 64-73%, while *tsb1-1* used in this study is a complete knockout. In this study, we found that not only *tsb1* mutants but also *trp3-1*, single substitution mutant of *TSA1*, exhibited open-stomata phenotype, whereas Liu et al. (2022) showed stress sensitivities in terms of chlorophyll content in *trp3-1* was similar to those in wild type. In addition, TSA1 did not interact with BG1 in yeast cells. These results suggest that TSB1 may affect stomatal aperture as well as TSA1 without affecting BG1.

Why does the *rtl2* mutant show an open-stomata phenotype compared to WT (**Figure 1**)? Jing et al. (2009) indicated that the *trp2* mutants showed higher auxin contents in plants. Ouyang et al. (2000) found that IAA contents in *TSA1* mutant, *trp3-1*, was also elevated and proposed Trp-independent IAA biosynthetic pathway, in which IAA is synthesized from IGP and indole. In this study, both *trp3-1* and *tsb1* mutants showed open-stomata phenotype (**Figure 5**). Stomatal phenotype in *trp3-1* and *tsb1* mutants showed correlation with IAA contents in these mutants. It has been demonstrated that auxin induces phosphorylation of the penultimate residue, Thr, of PM H^+^-ATPase and activation of PM H^+^-ATPase in the etiolated seedlings (Takahashi et al., 2012). To confirm the possibility that auxin mediates open-stomata phenotype in *rtl2*, we examined the effect of auxin on stomatal phenotypes. However, short-term treatment of auxin (within 3 hr) had no effect on stomatal aperture and phosphorylation status of PM H^+^-ATPase in guard cells (**Figure 6B, C**). Moreover, auxin antagonist auxinole did not affect the stomatal aperture in *tsb1* mutants (**Figure 6D**). Further investigations will be needed to investigate effect of long-term treatment of auxin (e.g., for several days) on stomatal opening and the phosphorylation level of PM H^+^-ATPase in guard cells. Exogenous application of Trp to *tsb1-1* resulted in suppression of open stomata phenotype in *tsb1-1* (**Figure 7B**). Although molecular mechanisms are still unclear, but to our knowledge, this is the first report that Trp biosynthetic pathway has significant effect on stomatal aperture in plants.

## Data availability statement

The original contributions presented in the study are included in the article/**Supplementary Materials**. Further inquiries can be directed to the corresponding author.

## Author contributions

MNS, YH and TK designed the experiments. MNS, YH, KT and TK performed experiments. MNS, YH and TK wrote the manuscript. All authors contributed to the article and approved the submitted version.

## Funding

This work was supported by Grants-in-Aid for Scientific Research from MEXT (nos. 20H05687 and 20H05910 to T.K.).

## Acknowledgments

We are grateful to Mami Uchida (Nagoya University) for technical support.

## Conflict of interest

The authors declare that the research was conducted in the absence of any commercial or financial relationships that could be construed as a potential conflict of interest.

## Publisher’s note

All claims expressed in this article are solely those of the authors and do not necessarily represent those of their affiliated organizations, or those of the publisher, the editors and the reviewers. Any product that may be evaluated in this article, or claim that may be made by its manufacturer, is not guaranteed or endorsed by the publisher.

## References

[B1] Akiyama M. Sugimoto H. Inoue S. I. Takahashi Y. Hayashi M. Hayashi Y. . (2022). Type 2C protein phosphatase clade D family members dephosphorylate guard cell plasma membrane H^+^-ATPase. Plant Physiol. 188, 2228–2240. doi: 10.1093/plphys/kiab571 34894269 PMC8968332

[B2] Ando E. Kinoshita T. (2018). Red light-induced phosphorylation of plasma membrane H^+^-ATPase in stomatal guard cells. Plant Physiol. 178, 838–849. doi: 10.1104/pp.18.00544 30104254 PMC6181031

[B3] Gibeaut D. M. Hulett J. Cramer R. Seemann J. R. (1997). Maximal biomass of arabidopsis fha/iana using a simple, low-maintenance hydroponic method and favorable environmental conditions. Plant Physiol. 115, 317–319. doi: 10.1104/pp.115.2.317 9342857 PMC158488

[B4] Greenberg J. B. Galston A. W. (1959). Tryptophan synthetase activity in pea seedling extracts. Plant Physiol. 34, 489–494. doi: 10.1104/pp.34.5.489 16655260 PMC541239

[B5] Hayashi M. Inoue S. I. Ueno Y. Kinoshita T. (2017). A raf-like protein kinase BHP mediates blue light-dependent stomatal opening. Sci. Rep. 7, 1–12. doi: 10.1038/srep45586 28358053 PMC5372365

[B6] Hayashi Y. Nakamura S. Takemiya A. Takahashi Y. Shimazaki K. I. Kinoshita T. (2010). Biochemical characterization of *in vitro* phosphorylation and dephosphorylation of the plasma membrane H^+^-ATPase. Plant Cell Physiol. 51, 1186–1196. doi: 10.1093/pcp/pcq078 20516032

[B7] Hayashi K. I. Neve J. Hirose M. Kuboki A. Shimada Y. Kepinski S. . (2012). Rational design of an auxin antagonist of the SCF TIR1 auxin receptor complex. ACS Chem. Biol. 7, 590–598. doi: 10.1021/cb200404c 22234040

[B8] Hayashi Y. Takahashi Y. Fukatsu K. Tada Y. Takahashi K. Kuwata K. . (2021). Identification of abscisic acid-dependent phosphorylated basic helix-Loop-Helix transcription factors in guard cells of *Vicia faba* by mass spectrometry. Front. Plant Sci. 12. doi: 10.3389/fpls.2021.735271 PMC8721282 34987530

[B9] Inoue S. I. Kinoshita T. (2017). Blue light regulation of stomatal opening and the plasma membrane H^+^-ATPase. Plant Physiol. 174, 531–538. doi: 10.1104/pp.17.00166 28465463 PMC5462062

[B10] Inoue S. I. Kinoshita T. Matsumoto M. Nakayama K. I. Doi M. Shimazaki K. I. (2008). Blue light-induced autophosphorylation of phototropin is a primary step for signaling. Proc. Natl. Acad. Sci. U. S. A. 105, 5626–5631. doi: 10.1073/pnas.0709189105 18378899 PMC2291087

[B11] Jing Y. Cui D. Bao F. Hu Z. Qin Z. Hu Y. (2009). Tryptophan deficiency affects organ growth by retarding cell expansion in arabidopsis. Plant J. 57, 511–521. doi: 10.1111/j.1365-313X.2008.03706.x 18980661

[B12] Kinoshita T. Doi M. Suetsugu N. Kagawa T. Wada M. Shimazaki K. (2001). phot1 and phot2 mediate blue light regulation of stomatal opening. Nature 414, 656–660. doi: 10.1038/414656a 11740564

[B13] Kinoshita T. Ono N. Hayashi Y. Morimoto S. Nakamura S. Soda M. . (2011). *FLOWERING LOCUS T* regulates stomatal opening. Curr. Biol. 21, 1232–1238. doi: 10.1016/j.cub.2011.06.025 21737277

[B14] Kinoshita T. Shimazaki K. I. (1999). Blue light activates the plasma membrane H^+^-ATPase by phosphorylation of the C-terminus in stomatal guard cells. EMBO J. 18, 5548–5558. doi: 10.1093/emboj/18.20.5548 10523299 PMC1171623

[B15] Last R. L. Bissinger P. H. Mahoney D. J. Radwanski E. R. Fink G. R. (1991). Tryptophan mutants in arabidopsis: The consequences of duplicated tryptophan synthase β genes. Plant Cell 3, 345–358. doi: 10.1105/tpc.3.4.345 1840915 PMC160005

[B16] Liu W.-C. Song R.-F. Zheng S.-Q. Li T.-T. Zhang B.-L. Gao X. . (2022). Coordination of plant growth and abiotic stress responses by tryptophan synthase β subunit 1 through modulation of tryptophan and ABA homeostasis in arabidopsis. Mol. Plant 15, 973–990. doi: 10.1016/j.molp.2022.04.009 35488429

[B17] Mashiguchi K. Tanaka K. Sakai T. Sugawara S. Kawaide H. Natsume M. . (2011). The main auxin biosynthesis pathway in arabidopsis. Proc. Natl. Acad. Sci. U. S. A. 108, 18512–18517. doi: 10.1073/pnas.1108434108 22025724 PMC3215075

[B18] Munemasa S. Hauser F. Park J. Waadt R. Brandt B. Schroeder J. I. (2015). Mechanisms of abscisic acid-mediated control of stomatal aperture. Curr. Opin. Plant Biol. 28, 154–162. doi: 10.1016/j.pbi.2015.10.010 26599955 PMC4679528

[B19] Ouyang J. Shao X. Li J. (2000). Indole-3-glycerol phosphate, a branchpoint of indole-3-acetic acid biosynthesis from the tryptophan biosynthetic pathway in *Arabidopsis thaliana* . Plant J. 24, 327–333. doi: 10.1046/j.1365-313X.2000.00883.x 11069706

[B20] Radwanski E. R. Barczak A. J. Last R. L. (1996). Characterization of tryptophan synthase alpha subunit mutants of *Arabidopsis thaliana* . Mol. Gen. Genet. 253, 353–361. doi: 10.1007/s004380050331 9003322

[B21] Radwanski E. R. Last R. L. (1995). Tryptophan biosynthesis and metabolism: Biochemical and molecular genetics. Plant Cell 7, 921–934. doi: 10.2307/3870047 7640526 PMC160888

[B22] Schroeder J. I. Allen G. J. Hugouvieux V. Kwak J. M. Waner D. (2001). Guard cell signal transduction. Annu. Rev. Plant Biol. 52, 627–658. doi: 10.1146/annurev.arplant.52.1.627 11337411

[B23] Schroeder J. I. Raschke K. Neher E. (1987). Voltage dependence of k^+^ channels in guard-cell protoplasts. Proc. Natl. Acad. Sci. U. S. A. 84, 4108–4112. doi: 10.1073/pnas.84.12.4108 16593851 PMC305032

[B24] Shimazaki K. I. Doi M. Assmann S. M. Kinoshita T. (2007). Light regulation of stomatal movement. Annu. Rev. Plant Biol. 58, 219–247. doi: 10.1146/annurev.arplant.57.032905.105434 17209798

[B25] Stepanova A. N. Hoyt J. M. Hamilton A. A. Alonso J. M. (2005). A link between ethylene and auxin uncovered by the characterization of two root-specific ethylene-insensitive mutants in arabidopsis. Plant Cell 17, 2230–2242. doi: 10.1105/tpc.105.033365 15980261 PMC1182485

[B26] Stepanova A. N. Robertson-Hoyt J. Yun J. Benavente L. M. Xie D. Y. Doležal K. . (2008). TAA1-mediated auxin biosynthesis is essential for hormone crosstalk and plant development. Cell 133, 177–191. doi: 10.1016/j.cell.2008.01.047 18394997

[B27] Takahashi K. Hayashi K. I. Kinoshita T. (2012). Auxin activates the plasma membrane H^+^-ATPase by phosphorylation during hypocotyl elongation in arabidopsis. Plant Physiol. 159, 632–641. doi: 10.1104/pp.112.196428 22492846 PMC3375930

[B28] Takemiya A. Kinoshita T. Asanuma M. Shimazaki K. I. (2006). Protein phosphatase 1 positively regulates stomatal opening in response to blue light in *Vicia faba* . Proc. Natl. Acad. Sci. U. S. A. 103, 13549–13554. doi: 10.1073/pnas.0602503103 16938884 PMC1569200

[B29] Takemiya A. Sugiyama N. Fujimoto H. Tsutsumi T. Yamauchi S. Hiyama A. . (2013). Phosphorylation of BLUS1 kinase by phototropins is a primary step in stomatal opening. Nat. Commun. 4, 2094. doi: 10.1038/ncomms3094 23811955

[B30] Tao Y. Ferrer J.-L. Ljung K. Pojer F. Hong F. Long J. A. . (2008). Rapid synthesis of auxin *via* a new tryptophan-dependent pathway is required for shade avoidance in plants. Cell 133, 164–176. doi: 10.1016/j.cell.2008.01.049 18394996 PMC2442466

[B31] Tivendale N. D. Ross J. J. Cohen J. D. (2014). The shifting paradigms of auxin biosynthesis. Trends Plant Sci. 19, 44–51. doi: 10.1016/j.tplants.2013.09.012 24524164

[B32] Toh S. Inoue S. Toda Y. Yuki T. Suzuki K. Hamamoto S. . (2018). Identification and characterization of compounds that affect stomatal movements. Plant Cell Physiol. 59, 1568–1580. doi: 10.1093/pcp/pcy061 29635388

[B33] Toh S. Takata N. Ando E. Toda Y. Wang Y. Hayashi Y. . (2021). Overexpression of plasma membrane H^+^-ATPase in guard cells enhances light-induced stomatal opening, photosynthesis, and plant growth in hybrid aspen. Front. Plant Sci. 12. doi: 10.3389/fpls.2021.766037 PMC8663642 34899787

[B34] Tsuzuki T. Takahashi K. Inoue S.i. Okigaki Y. Tomiyama M. Hossain M. A. . (2011). Mg-chelatase H subunit affects ABA signaling in stomatal guard cells, but is not an ABA receptor in *Arabidopsis thaliana* . J. Plant Res. 124, 527–538. doi: 10.1007/s10265-011-0426-x 21562844 PMC3129500

[B35] Tzin V. Galili G. (2010). The biosynthetic pathways for shikimate and aromatic amino acids in *Arabidopsis thaliana* . Arab. B. 8, e0132. doi: 10.1199/tab.0132 PMC3244902 22303258

[B36] Uchida N. Takahashi K. Iwasaki R. Yamada R. Yoshimura M. Endo T. A. . (2018). Chemical hijacking of auxin signaling with an engineered auxin-TIR1 pair. Nat. Chem. Biol. 14, 299–305. doi: 10.1038/nchembio.2555 29355850 PMC5812785

[B37] Ursache R. Miyashima S. Chen Q. Vatén A. Nakajima K. Carlsbecker A. . (2014). Tryptophan-dependent auxin biosynthesis is required for HD-ZIP III-mediated xylem patterning. Dev. 141, 1250–1259. doi: 10.1242/dev.103473 PMC7055496 24595288

[B38] Wang B. Chu J. Yu T. Xu Q. Sun X. Yuan J. . (2015). Tryptophan-independent auxin biosynthesis contributes to early embryogenesis in arabidopsis. Proc. Natl. Acad. Sci. U. S. A. 112, 4821–4826. doi: 10.1073/pnas.1503998112 25831515 PMC4403211

[B39] Wang Y. Noguchi K. Ono N. Inoue S. I. Terashima I. Kinoshita T. (2014). Overexpression of plasma membrane H^+^-ATPase in guard cells promotes light-induced stomatal opening and enhances plant growth. Proc. Natl. Acad. Sci. U. S. A. 111, 533–538. doi: 10.1073/pnas.1305438111 24367097 PMC3890815

[B40] Wang Y. Noguchi K. Terashima I. (2011). Photosynthesis-dependent and -independent responses of stomata to blue, red and green monochromatic light: Differences between the normally oriented and inverted leaves of sunflower. Plant Cell Physiol. 52, 479–489. doi: 10.1093/pcp/pcr005 21257606

[B41] Ye W. Munemasa S. Shinya T. Wu W. Ma T. Lu J. . (2020). Stomatal immunity against fungal invasion comprises not only chitin-induced stomatal closure but also chitosan-induced guard cell death. Proc. Natl. Acad. Sci. U. S. A. 117, 20932–20942. doi: 10.1073/pnas.1922319117 32778594 PMC7456093

[B42] Zhang M. Wang Y. Chen X. Xu F. Ding M. Ye W. . (2021). Plasma membrane H^+^-ATPase overexpression increases rice yield *via* simultaneous enhancement of nutrient uptake and photosynthesis. Nat. Commun. 12. doi: 10.1038/s41467-021-20964-4 PMC7854686 33531490

[B43] Zhao Y. (2012). Auxin biosynthesis: A simple two-step pathway converts tryptophan to indole-3-Acetic acid in plants. Mol. Plant 5, 334–338. doi: 10.1093/mp/ssr104 22155950 PMC3309920

