# Supplementary material for: Tryptophan synthase ß subunit 1 affects stomatal phenotypes in Arabidopsis thaliana
Source: Front Plant Sci. 2022 Nov 28;13:1011360. doi: 10.3389/fpls.2022.1011360 (PMC9743989; doi:10.3389/fpls.2022.1011360)
Supplement: Supplementary file 1 [file DataSheet_1.pdf]

Supplementary Figure 1

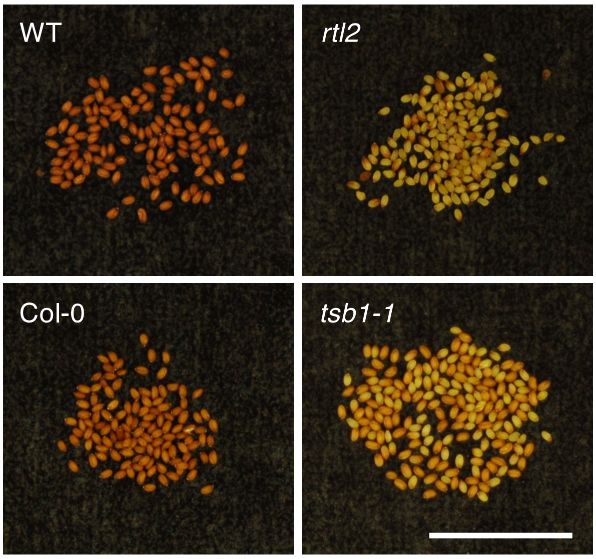

SUPPLEMENTARY FIGURE 1 Color of dry seeds from WT, *rtl2*, Col-0, and *tsb1-1*. Dry seeds from mutants showed a lighter color than background strain seeds. Scale bar = 5 mm.
