# Supplementary material for: Tryptophan synthase ß subunit 1 affects stomatal phenotypes in Arabidopsis thaliana
Source: Front Plant Sci. 2022 Nov 28;13:1011360. doi: 10.3389/fpls.2022.1011360 (PMC9743989; doi:10.3389/fpls.2022.1011360)
Supplement: Supplementary file 2 [file DataSheet_2.pdf]

Supplementary Figure 2

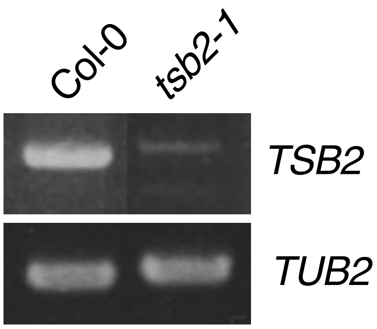

SUPPLEMENTARY FIGURE 2 *TSB2* expression analyzed by RT-PCR in Col-0 and *tsb2-1*. Total RNA was extracted from rosette leaves of 5-week-old plants grown on MS plate for 4 weeks and transferred to soil for 1 week. *TUB2* was amplified as a control. PCRs were performed with 30 cycles for *TSB2* and with 25 cycles for *TUB2*, respectively.
