# Supplementary material for: Tryptophan synthase ß subunit 1 affects stomatal phenotypes in Arabidopsis thaliana
Source: Front Plant Sci. 2022 Nov 28;13:1011360. doi: 10.3389/fpls.2022.1011360 (PMC9743989; doi:10.3389/fpls.2022.1011360)
Supplement: Supplementary file 3 [file Table_1.pdf]

SUPPLEMENTARY TABLE 1. Effect of exogenous L-Trp on stomatal size, index and density of *tsb1-1*.

		Stomatal size (μm)	Stomatal index	Stomatal density (mm^{-2})
Mock	Col-0	20.5 ± 2.31	0.212 ± 0.031	213 ± 35.9
	tsb1-1	20.3 ± 2.39	0.194 + 0.021	167 ± 92.6
Trp	Col-0	21.0 ± 2.57	0.268 + 0.051	239 ± 68.5
	tsb1-1	21.3 ± 2.49	0.211 + 0.013	194± 10.1

Ten-day-old seedlings germinated on MS plate were transferred to hydroponic system with or without 0.25 mM L-Trp, and were grown for a further 3 to 4 weeks. Other details are the same as in Table 1.
