# Supplementary material for: Tryptophan synthase ß subunit 1 affects stomatal phenotypes in Arabidopsis thaliana
Source: Front Plant Sci. 2022 Nov 28;13:1011360. doi: 10.3389/fpls.2022.1011360 (PMC9743989; doi:10.3389/fpls.2022.1011360)
Supplement: Supplementary file 4 [file Table_2.pdf]

SUPPLEMENTARY TABLE 2. List of primers for RT-PCR and qRT-PCR

RT-PCR		
Gene	Fw/Rv	Sequence (5’ to 3’)
TSB1	Fw	TTCAGTAATGGCAGCCTCAGG
	Rv	TCAAACATCAAGATATTTAGCCACTGTC
TSB2	Fw	ATGGCCACCGCAAGCAC
	Rv	TTAAACTTCGAGATACTTGATTGCAGTC
TUB2	Fw	TTCACTCATCCAACCGAAGATGC
	Rv	CAACTGGTAGTTGAGGTGTTCC
qRT-PCR		
Gene	Fw/Rv	Sequence (5’ to 3’)
AUR3	Fw	ATGTTCTCCTGGTTAGCGAGAATGG
	Rv	CTAAGAGAGGAAGATACTGAACTTG
SAUR9	Fw	TCAACACCGAAGTCGCTATG
	Rv	TCGTGCTCGAAACCAAACTC
IAA1	Fw	TGGACGGAGCTCCATATCTCC
	Rv	CACCGACCAACATCCAATCTCC
IAA24	Fw	GCCTAGTTAGTGTTGAGTGTGGGA
	Rv	CGGCTTTCTTGTACCTGACTGG
TUB2	Fw	GAATATCAACAGGAGGAAGAGTACTGAG
	Rv	AACTACAACTGGTAGTTGAGGTGTTCC
